# Metabolic disorders and risk of cardiovascular diseases: a two-sample mendelian randomization study

**DOI:** 10.1186/s12872-023-03567-3

**Published:** 2023-10-31

**Authors:** Zhe Wang, Jiawei Chen, Longyang Zhu, Siqi Jiao, Yinong Chen, Yihong Sun

**Affiliations:** 1 Department of Cardiology, China-Japan Friendship Hospital (Institute of Clinical Medical Sciences), Chinese Academy of Medical Sciences & Peking Union Medical College, Beijing, China; 2 Department of Cardiology, The First Affiliated Hospital of Zhengzhou University, Zhengzhou, Henan China; 3 Department of Cardiology, Peking University China-Japan Friendship School of Clinical Medicine, Beijing, China; 4 Department of Cardiology, China-Japan Friendship Hospital, No.2 East Yinghua Road, Chaoyang District, Beijing, 100029 China

**Keywords:** Metabolic disorders, Cardiovascular diseases, Mendelian randomization analysis

## Abstract

**Background:**

Metabolic disorders are increasing worldwide and are characterized by various risk factors such as abdominal obesity, insulin resistance, impaired glucose metabolism, and dyslipidemia. Observational studies suggested a bidirectional association between cardiovascular diseases and metabolic disorders and its components. However, the causal associations between them remained unclear. This study aims to investigate the causal relationship between metabolic disorders and cardiovascular disease through Mendelian randomization (MR) analysis.

**Methods:**

A two-sample MR analysis based on publicly available genome-wide association studies were used to infer the causality. The single-nucleotide polymorphisms with potential pleiotropy were excluded by MR-PRESSO. The effect estimates were constructed using the random-effects inverse-variance-weighted method as the primary estimate. Furthermore, MR-Egger and weighted median were also performed to detect heterogeneity and pleiotropy.

**Results:**

Genetically predicted metabolic disorders increased the risk for coronary heart disease (OR = 1.77, 95% CI: 1.55–2.03, p < 0.001), myocardial infarction (OR = 1.75, 95% CI: 1.52–2.03, p < 0.001), heart failure (OR = 1.26, 95% CI: 1.14–1.39, p < 0.001), hypertension (OR = 1.01, 95% CI: 1.00-1.02, p = 0.002), and stroke (OR = 1.19, 95% CI: 1.08–1.32, p < 0.001). The concordance of the results of various complementary sensitivity MR methods reinforces the causal relationship further.

**Conclusion:**

This study provides evidence of a causal relationship between metabolic disorders and increased risk of coronary heart disease, myocardial infarction, heart failure, hypertension, and stroke. Special attention should be paid to improving metabolic disorders to reduce the development of cardiovascular diseases.

**Supplementary Information:**

The online version contains supplementary material available at 10.1186/s12872-023-03567-3.

## Introduction

Metabolic disorders are metabolic syndromes that combine physiological, biochemical, and clinical factors, mainly insulin resistance, visceral fat accumulation, dyslipidemia, endothelial dysfunction, and so on [1]. Cardiovascular comorbidities and outcomes have historically been underestimated in metabolic disorders. Recently, a significant increase in co-morbidity incidence between metabolic disorders and cardiovascular disease has been observed [2]. Coronary heart disease (CHD) represents a major global health burden, and 58% of patients with CHD have metabolic syndrome [3]. One study revealed a clear dose-response relationship between cumulative exposure to metabolic disorders and myocardial infarction (MI) and the importance of metabolic disorders management for preventing cardiovascular diseases [4]. Another study revealed a positive association between cumulative metabolic burdens and the risk of developing atrial fibrillation (AF) [5]. Encouraging an active lifestyle can improve some patients’ metabolic disorders and help to control blood pressure [6]. Individual components of metabolic syndrome or metabolic syndrome increase the risk of heart failure (HF) and ischemic stroke [7].

Confounding factors and reverse causality bias could not be adequately accounted for in observational studies, and therefore the causal relationship between metabolic disorders and different cardiovascular diseases remain uncertain [8]. It is worth investigating the causal relationship between metabolic disorders and cardiovascular diseases outcome. The availability of large-scale genome-wide association studies (GWASs) enables the search for causal relationships [9]. Mendelian randomization (MR) analysis can overcome the reverse causality bias, as randomization of alleles always precedes the onset of cardiovascular diseases. The independent assessment of random distributions and genetic polymorphisms allowed the MR analysis to decrease the effect of confounding factors by introducing genetic markers as instrumental variables (IVs) for exposure factors [10]. Our study aimed to comprehensively investigate the association of genetically predicted metabolic disorders with cardiovascular diseases by two samples of MR analysis.

## Methods

### Study design

**Fig. 1:**
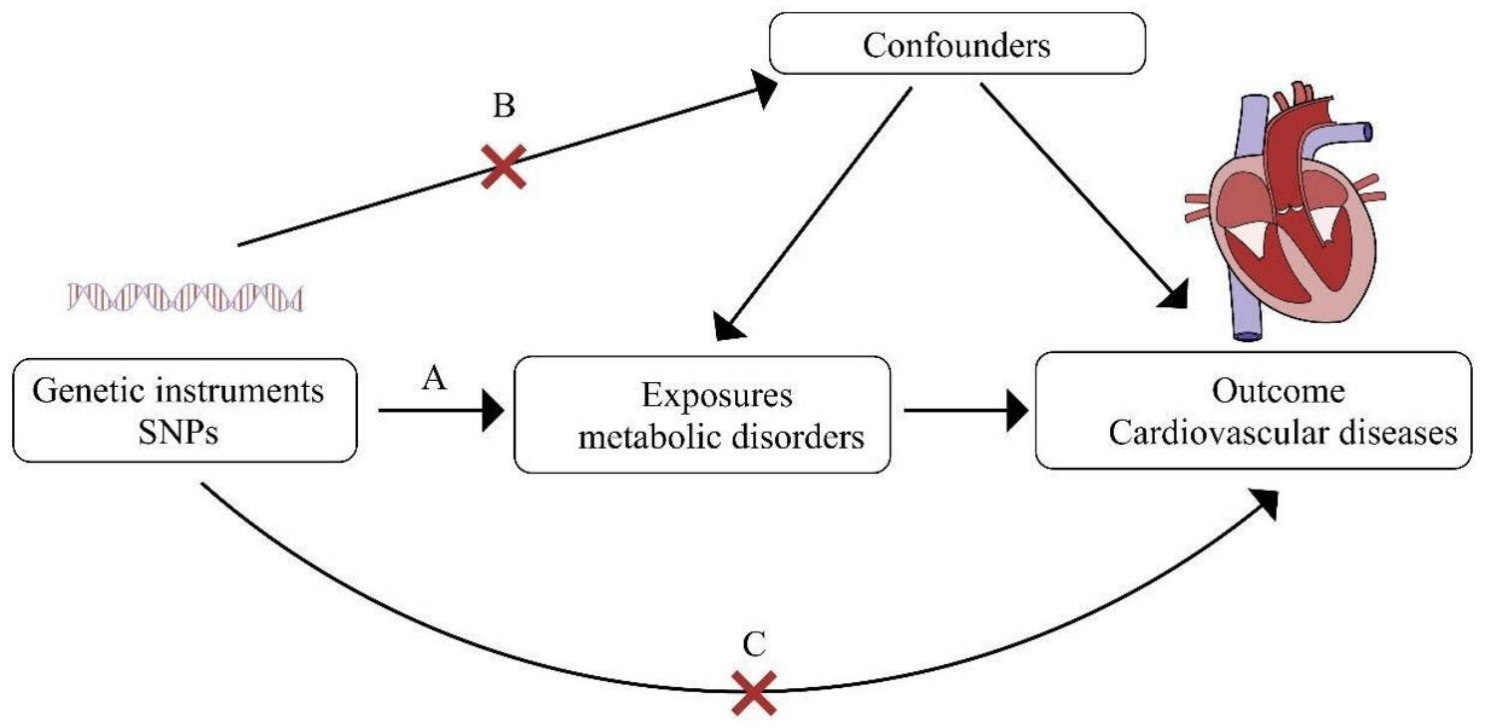
Three critical assumptions of the Mendelian randomization study. **A**: SNPs are strongly associated with metabolic disorders; **B**: The selected SNPs are not associated with confounding factors; **C**: SNPs affect cardiovascular diseases only through metabolic disorders. SNPs, single-nucleotide polymorphisms

A schematic diagram of the study design, along with the three key assumptions of MR, as shown in Fig. 1. As follows: (A) single nucleotide polymorphisms (SNPs) are strongly associated with metabolic disorders; (B) selected genes are not associated with confounding factors; (C) SNPs affect cardiovascular diseases only through metabolic disorders.

### Data sources

The analysis was performed utilizing published summary-level data from GWASs of the traits of interest in European individuals. GWAS summary statistics for metabolic disorders (21,533 cases and 197,259 controls of Finnish descent) were obtained from the FinnGen Consortium, which evaluated the association between the number of metabolic disorders and SNPs [11]. The FinnGen study is a unique study that combines genomic information with digital medical data from participants over 18 years of age living in Finland [12]. The resource includes prospective epidemiological cohorts, disease-based cohorts, and hospital biobank samples. Detailed information can be accessed on the official website (https://www.finngen.fi/fi). Information on diagnoses of cardiovascular disorders (International Classification of Diseases-Tenth Edition [ICD-10], codes E [7-9] were ascertained from the national Finland hospital discharge register and the causes of death register.

Summary statistics for AF were obtained from the public GWAS) pooled in the European Bioinformatics Institute (EBI) database. The populations of European origin were used in our study, which consisted of 60,620 cases and 970,216 control participants [13]. CHD (60,801 cases and 123,504 controls) and MI (43,676 cases and 128,199 controls) were obtained from a meta-analysis of coronary artery disease [14]. HF was obtained from a GWAS conducted in 26 studies of the Heart Failure Therapeutic Target Molecular Epidemiology (HERMES) consortium, including 47,309 cases and 930,014 controls of European ancestry [15]. Summary statistics for genetic associations with hypertension (54,358 cases and 408,652 controls) were derived from imputed genotype data from the UK Biobank, which the results reported by Van Oort., et al. [16] Stroke (40,585 cases and 406,111 controls) was acquired from results reported by Malik, R., et al. [17].

### Ethics

A publicly available and identifiable database was used for this study. These data were obtained from participant studies approved by the Ethical Standards Committee. No separate ethical approval is required for this study.

### SNPs selection and validation

We applied three criteria to select eligible SNPs. We conducted a rigorous selection procedure for SNPs, which may reduce the ability to explain phenotypic variation in metabolic disorders. First, we selected SNPs related to metabolic disorders with a genome-wide significance threshold of p < 5 × 10^− 8^. Second, The PLINK clumping method was used to identify independent SNPs. To minimize the linkage disequilibrium (LD), which can bias the randomized assignment results. A stringent condition (LD threshold of r^2^ < 0.001, the distance of 10 000 kb from each other) was used to ensure that the genetic tools selected for metabolic disorders, which were conditionally independent. Third, F-statistics were counted to verify the strength of each SNP. SNPs were considered strong enough to mitigate the effects of potential bias when F-statistics were greater than 10. The data harmonization step was performed before MR analysis, as the effect of SNPs on exposure and outcome must correspond to the same allele. Moreover, SNPs with potential pleiotropy were excluded after MR-pleiotropy residual sum and outlier (MR-PRESSO), and MR analysis was performed to evaluate the robustness. We also checked SNPs in PhenoScanner (www.phenoscanner.medschl.cam.ac.uk), a platform with comprehensive information on the association between genotype and phenotype.

### Statistical methods

SNPs related to cardiovascular diseases outliers established by MRPRESSO were excluded. Three different MR methods random effects inverse variance weighted (IVW), MR Egger, and weighted median were applied to fix the heterogeneity and polymorphism effects of the variants. IVW was applied as the main outcome, while MR-Egger and weighted median were used to improve the IVW estimates as they could provide more robust estimates over a wider range of situations. The weighted median method can offer a valid estimate when more than 50% of the information is from valid IVs [18]. The MR-Egger method can be performed to evaluate the horizontal polymorphism of the selected IVs [19]. Cochrane’s Q-test was performed to identify heterogeneity among selected IVs. A funnel plot was applied to estimate possible directional variability, similar to the assessment of publication bias in meta-analysis. For significant estimates, we further assessed horizontal pleiotropy, utilizing the MR-Egger intercept test and leave-one-out analyses, which determine whether the overall estimate is disproportionately influenced by individual SNPs. To take into account multiple testing for metabolic disorders and cardiovascular disease, a Bonferroni-corrected threshold of p < 0.008 (α = 0.05/6) was applied separately for cardiovascular diseases outcome. When the p-value was between the Bonferroni-corrected value and 0.05, it was regarded as nominally significant, which was considered suggestive of evidence of association. All statistical analyses were performed using the “TwoSampleMR” packages in R version 4.2.3 (R Foundation for Statistical Computing, Vienna, Austria).

## Results

### SNP selection and validation

In summary, 14 IVs achieved genome-wide significance levels, and all F-statistics were greater than 10 (Supplementary Table 1). The 14 IVs for metabolic disorders selected mainly represented dyslipidemia and obesity. Some outlier SNPs were detected for some cardiovascular diseases in the MR-PRESSO analysis. 3 outlier SNPs (rs13032842, rs646776, rs9644859) for metabolic disorders and CHD, 3 outlier SNPs (rs11591147, rs13032842, rs9644859) for metabolic disorders and MI, 4 outlier SNPs (rs1367117, rs646776, rs7412, rs9644859) for metabolic disorders and HF, 1 outlier SNP (rs9644859) for metabolic disorders and hypertension, 1 outlier SNP (rs9644859) for metabolic disorders and stroke were excluded in our final MR analysis.

### Cardiovascular diseases

**Fig. 2:**
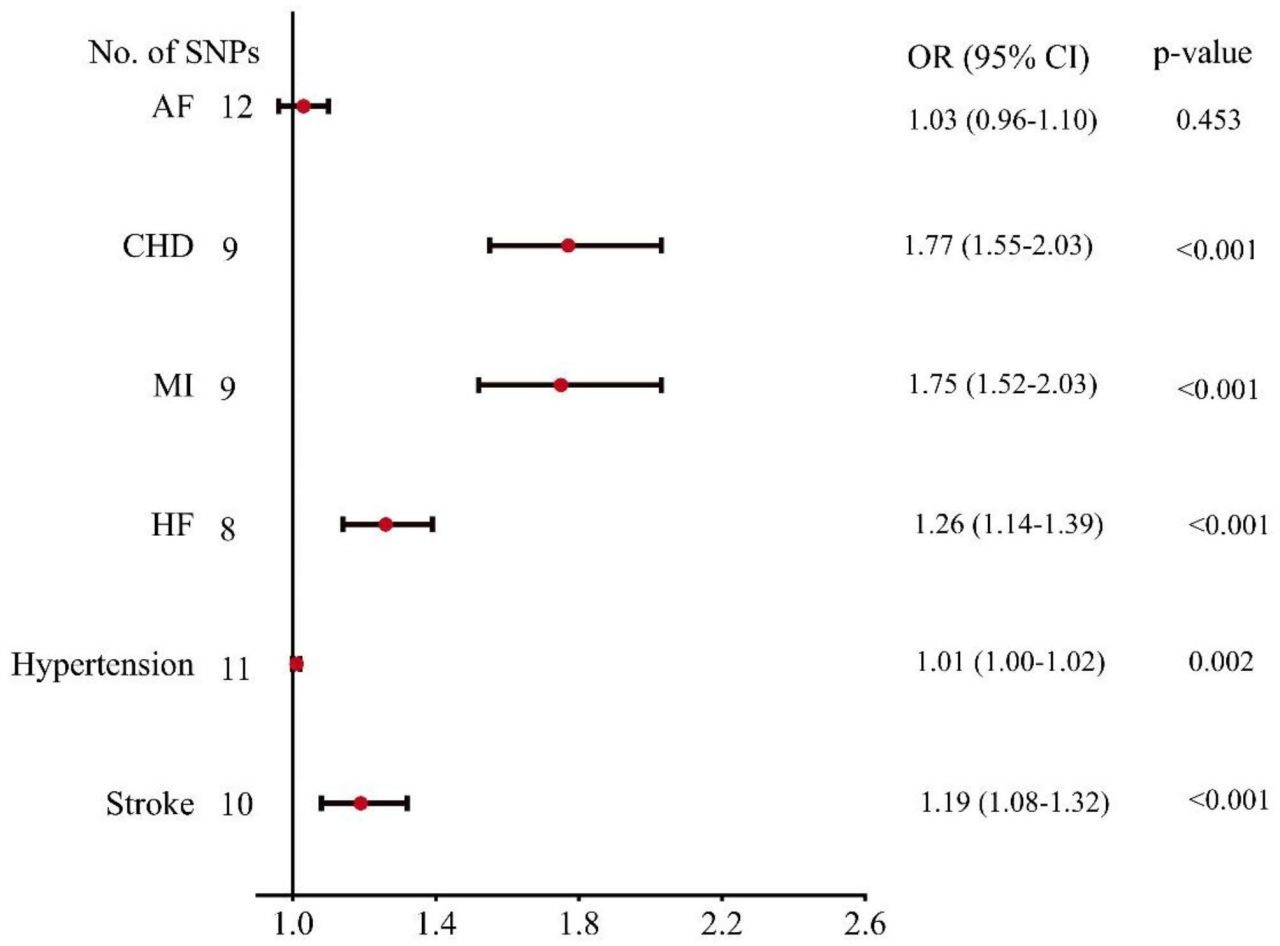
Associations of genetically predicted metabolic disorders with cardiovascular diseases except for outlier SNPs. AF, atrial fibrillation; CHD, coronary heart disease; CI, confidence interval; MI, myocardial infarction; HF, heart failure; OR, odds ratio; SNPs, single-nucleotide polymorphisms

Random-effects IVW analysis indicated that each standard deviation increase in genetically predicted metabolic disorders was related to four cardiovascular diseases, including CHD (OR = 1.77, 95% CI: 1.55–2.03, p < 0.001), MI (OR = 1.75, 95% CI: 1.52–2.03, p < 0.001), HF (OR = 1.26, 95% CI: 1.14–1.39, p < 0.001), hypertension (OR = 1.01, 95% CI: 1.00-1.02, p = 0.002), and stroke (OR = 1.19, 95% CI: 1.08–1.32, p < 0.001). However, no associations were observed for metabolic disorders and AF (OR = 1.03, 95% CI: 0.96–1.10, p = 0.453), as depicted in Fig. 2.

**Fig. 3:**
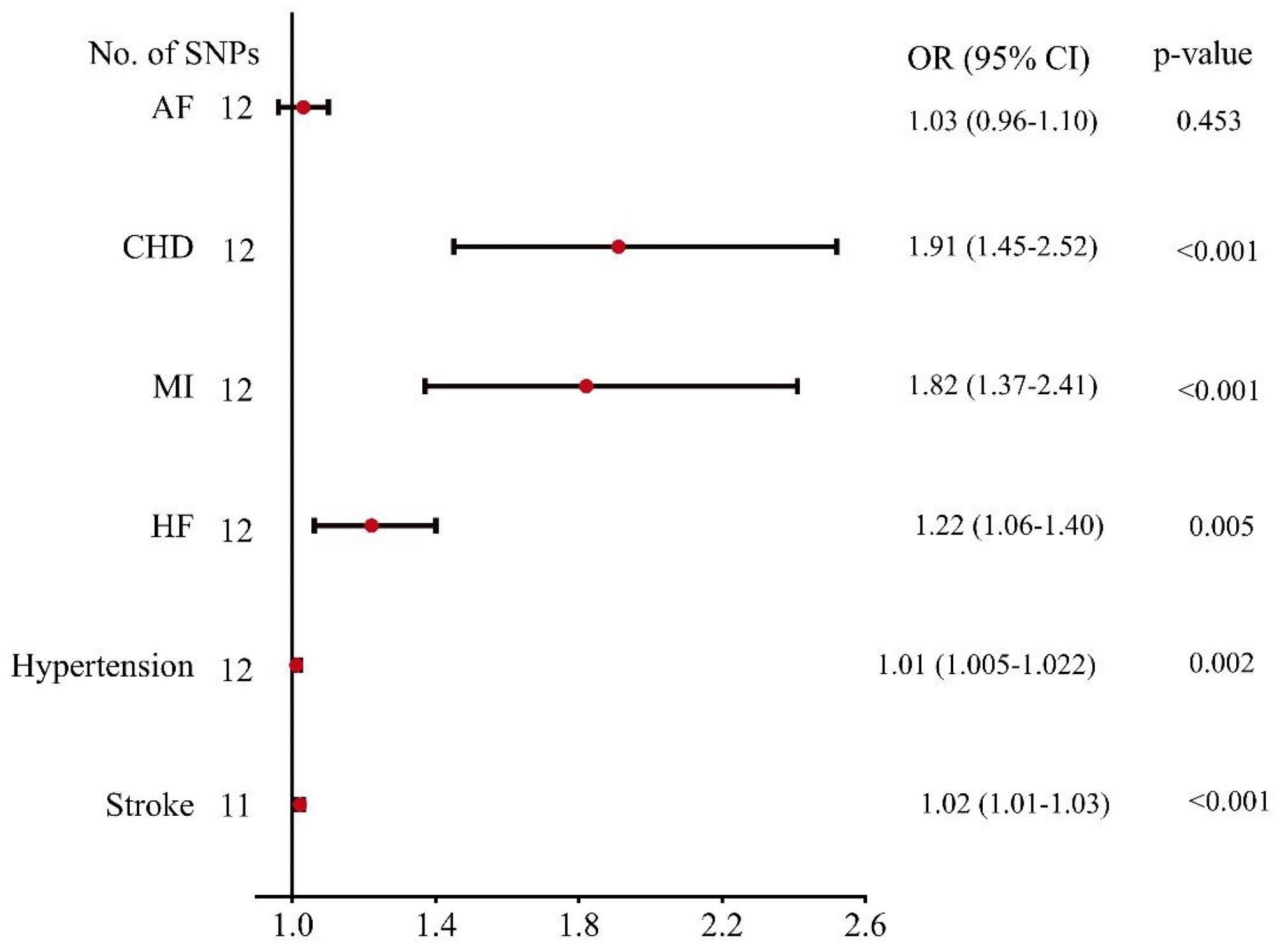
Associations of genetically predicted metabolic disorders with cardiovascular diseases include outlier SNPs. AF, atrial fibrillation; CHD, coronary heart disease; CI, confidence interval; MI, myocardial infarction; HF, heart failure; OR, odds ratio; SNPs, single-nucleotide polymorphisms

The weighted median analysis equally observed the causal association between metabolic disorders and some cardiovascular diseases. However, we did not observe evidence of the causal association between metabolic disorders and HF using the MR-Egger analysis described in Table 1. Furthermore, we revealed no evidence of directional pleiotropy. The heterogeneity was observed for some cardiovascular diseases, as shown in Table 2. Furthermore, when outlier SNPs were included in our study, the random-effects IVW analysis also found a similar causal relationship between metabolic disorders and cardiovascular diseases outcome, as shown in Fig. 3.

**Table 1:** Associations between genetically predicted metabolic disorders and cardiovascular diseases in the sensitivity analysis using weighted-median and MR-Egger methods

Outcome	Weighted Median			MR-Egger	
	OR (95% CI)	p-value		OR (95% CI)	p-value
AF	1.03 (0.95–1.11)	0.494		1.04 (0.89–1.22)	0.643
CHD	1.73 (1.50-2.00)	< 0.001		1.94 (1.43–2.64)	0.004
MI	1.75 (1.52–2.02)	< 0.001		1.71 (1.17–2.48)	0.027
HF	1.34 (1.18–1.51)	< 0.001		1.23 (0.91–1.66)	0.230
Hypertension	1.01 (1.01–1.02)	< 0.001		1.02 (1.01–1.04)	0.021
Stroke	1.19 (1.06–1.33)	0.003		1.34 (1.07–1.69)	0.031

Abbreviations: AF, atrial fibrillation; CHD, coronary heart disease; CI, confidence interval; MI, myocardial infarction; HF, heart failure; OR, odds ratio

**Table 2:** Associations between genetically predicted metabolic disorders and cardiovascular disease in the sensitivity analysis using pleiotropy and heterogeneity

Outcome	Pleiotropy			Heterogeneity	
	Intercept	p-value		Q	p-value
AF	-0.001	0.859		10	0.071
CHD	-0.010	0.531		7	0.014
MI	-0.003	0.877		7	0.008
HF	0.002	0.866		6	0.181
Hypertension	-0.001	0.186		9	0.070
Stroke	-0.013	0.285		10	0.060

Abbreviations: AF, atrial fibrillation; CHD, coronary heart disease; MI, myocardial infarction; HF, heart failure

Scatter plots, forest plots, and the funnel plot of the association between metabolic disorders and cardiovascular diseases are shown in Supplementary Figs. 1–2, respectively, where similar results can be observed. The leave-one-out sensitivity analysis is shown in Supplementary Figs. 3–7, which revealed that the overall estimates were not disproportionately affected by any individual SNP.

## Discussion

In the two-sample MR analysis, we assessed the causal relationship between metabolic disorders and cardiovascular diseases. Our results found that metabolic disorders were positively associated with CHD, MI, HF, hypertension, and stroke. Moreover, our results are primarily robust to several sensitivity analyses. Detecting and correcting metabolic disorders before they develop, when possible, may be important to prevent the development of cardiovascular diseases.

Disruption of normal metabolic processes leads to energy and redox imbalances planting the seeds for many pathophysiological conditions in the body which are collectively labeled as metabolic disorders [20]. A comprehensive MR study revealed that the causal link between homocysteine and increased metabolic disorders risk [21]. Another MR study found that genetically predicted short sleep duration is a potentially causal risk factor for metabolic syndrome [22]. Recent studies also revealed that abdominal obesity, impaired glucose metabolism, dyslipidemia, high uric acid, and insulin resistance are metabolite biomarkers for metabolism disorders [23]. Recent reports have drawn attention to the cardiovascular aspects of metabolic disorders [2]. Up to date, we consider that direct evidence to estimate the causal relationship between metabolic disorders and cardiovascular diseases is still insufficient.

There have been a number of large cohort studies investigating the risk of metabolic disorders with cardiovascular diseases [24]. The prevalence of metabolic syndrome is estimated to be about 20–25% of the population [25]. People with metabolic syndrome are twice as likely to die from CHD and three times as likely to suffer a heart attack or stroke compared to people without metabolic syndrome [26]. Metabolic disorders are common in chronic HF and independently related to increased all-cause mortality [27]. Our study revealed that metabolic disorders causally influence CHD, MI, HF, hypertension, and stroke, suggesting metabolic disorders play an essential role in primary and secondary cardiovascular disease prevention. An observational study found a positive association between cumulative metabolic burden and the risk of developing AF [5]. However, our study did not reveal that metabolic disorders contribute to the development of AF by MR analysis. The effect of metabolic disorders in observational studies may be inaccurate. In contrast to previous meta-analyses based mainly on observational studies, our results from MR analysis may provide a more solid conclusion, MR analysis is not influenced by confounding factors or reverse causality.

The statistical power of the random effect IVW method is obviously superior to the statistical power of other MR methods, especially MR-Egger [28]. MR-Egger results with low statistical power had broader reliability and non-significant p-values compared to random effect IVW. IVW is commonly adopted as the main method to screen for potentially important outcomes. Other MR methods were performed to secure the robustness of IVW [29]. Due to we used the random effects of IVW as the primary results, heterogeneity is acceptable [30]. The preference for fixed IVW in the absence of heterogeneity in study findings. In the presence of heterogeneity in the study findings, we used randomized IVW results to be more stable. The IVW analysis under a random-effects model was performed to alleviate the impacts of heterogeneity [29, 31]. Moreover, the researchers have strengthened the requirement for consistent beta direction in all MR analysis methods, which was used in our study [32]. Our study ensures the accuracy of MR analysis by passing Bonferroni correction.

The underlying pathophysiological mechanism between metabolic disorders and cardiovascular diseases has not yet been fully established. Several critical mechanisms can be used to illustrate this regulation. First, the increased systemic oxidative stress is closely related to metabolic disorders [33]. Reactive oxygen species (ROS) generated in metabolic disorders could contribute to the development of cardiovascular damage by upregulating redox signaling pathways and changing the gene expression of inflammatory cytokines, chemokines, and growth factors [34]. Besides, Lipid accumulation is one of the manifestations of metabolic disorders, which may lead to apoptosis, impaired mitochondrial function, cardiac hypertrophy, and contractile dysfunction [35]. The increased cardiac lipid accumulation and altered substrate metabolism could alter the hemodynamic load and cause cardiovascular complications [36]. Moreover, metabolic disorders could induce endoplasmic reticulum stress, which may distort the equilibrium of free radical productions and antioxidant capability, resulting in cardiac stress [37]. Future researches are needed to explore the potential mechanisms, which is essential for developing relevant clinical recommendations.

Although the findings are significant, understanding the limitations of study means a better interpretation of the findings. First, since MR analyses inferred causal hypotheses by utilizing the random allocation of genetic variants, it is hard to fully discriminate between mediation and pleiotropy using MR methods. The generous variants in our genome potentially influence one or more phenotypes. Second, the two-sample MR study was based on European ancestries, which increased ancestry bias. Future studies on other ancestries are needed. Furthermore, we did not have specific subtypes of metabolic disorders and insufficient power to detect different levels of metabolic disorders with cardiovascular diseases, which limits our ability to perform further analysis. Future studies will investigate the causal relationship between specific phenotypes of metabolic disorders and cardiovascular diseases.

## Conclusions

This study provides evidence of a positive causal relationship between metabolic disorders and increased cardiovascular diseases risk of CHD, MI, HF, hypertension, and stroke. Metabolic disorders play an active role in promoting the progression of cardiovascular diseases, highlighting the importance of revealing the underlying mechanisms for intervention targets.

### Electronic supplementary material

Below is the link to the electronic supplementary material.

Supplementary Material 1

## Data Availability

Only publicly available GWAS summary data were used in this work. All raw data for this study are publicly available in the IEU Open GWAS Project repository (http://gwas.mrcieu.ac.uk). Metabolic disorders summary data are available on the respective websites (https://gwas.mrcieu.ac.uk/datasets/finn-b-E4_METABOLIA). Atrial fibrillation summary data are available on the respective websites (https://gwas.mrcieu.ac.uk/datasets/ebi-a-GCST006414). Coronary heart disease summary data are available on the respective websites (https://gwas.mrcieu.ac.uk/datasets/ieu-a-7). Myocardial infarction summary data are available on the respective websites (https://gwas.mrcieu.ac.uk/datasets/ieu-a-798). Heart failure summary data are available on the respective websites (https://gwas.mrcieu.ac.uk/datasets/ebi-a-GCST009541). Hypertension summary data are available on the respective websites (https://gwas.mrcieu.ac.uk/datasets/ukb-b-12493). Stroke summary data are available on the respective websites (https://gwas.mrcieu.ac.uk/datasets/ebi-a-GCST006906). All findings are reproducible.
