# Supplementary material for: Metabolic disorders and risk of cardiovascular diseases: a two-sample mendelian randomization study
Source: BMC Cardiovasc Disord. 2023 Oct 31;23:529. doi: 10.1186/s12872-023-03567-3 (PMC10617200; doi:10.1186/s12872-023-03567-3)
Supplement: Supplementary file 1 — Supplementary Material 1 [file 12872_2023_3567_MOESM1_ESM.docx]

**Supplementary Table 1.** SNPs used as instrumental variables in the Mendelian randomization analyses of metabolic disorders.

| SNP | Chr | EA | OA | Eaf | p-value | Beta | SE | F |
| --- | --- | --- | --- | --- | --- | --- | --- | --- |
| rs499883 | 1 | A | G | 0.529 | 4.67E-09 | 0.064 | 0.011 | 441 |
| rs11591147 | 1 | T | G | 0.036 | 1.83E-18 | -0.260 | 0.030 | 1035 |
| rs646776 | 1 | T | C | 0.785 | 1.95E-10 | 0.084 | 0.013 | 518 |
| rs182549 | 2 | T | C | 0.597 | 1.54E-09 | -0.067 | 0.011 | 468 |
| rs13032842 | 2 | G | A | 0.158 | 8.79E-09 | -0.086 | 0.015 | 427 |
| rs1367117 | 2 | A | G | 0.281 | 2.16E-08 | 0.067 | 0.012 | 395 |
| rs4299376 | 2 | T | G | 0.783 | 2.65E-09 | -0.078 | 0.013 | 453 |
| rs77645768 | 4 | A | G | 0.027 | 3.10E-08 | 0.187 | 0.034 | 401 |
| rs2954021 | 8 | G | A | 0.536 | 6.35E-14 | -0.081 | 0.011 | 711 |
| rs9644859 | 9 | A | G | 0.418 | 2.11E-09 | 0.065 | 0.011 | 454 |
| rs115478735 | 9 | T | A | 0.198 | 7.55E-11 | 0.088 | 0.014 | 536 |
| rs964184 | 11 | C | G | 0.854 | 8.74E-14 | -0.114 | 0.015 | 707 |
| rs7412 | 19 | T | C | 0.054 | 1.18E-24 | -0.250 | 0.024 | 1392 |
| rs118068660 | 19 | T | C | 0.103 | 8.08E-19 | -0.159 | 0.018 | 1021 |

Abbreviations: Chr, chromosome; EA, non-effect allele; OA, other alleles; SE, standard error; SNP, single-nucleotide polymorphism.


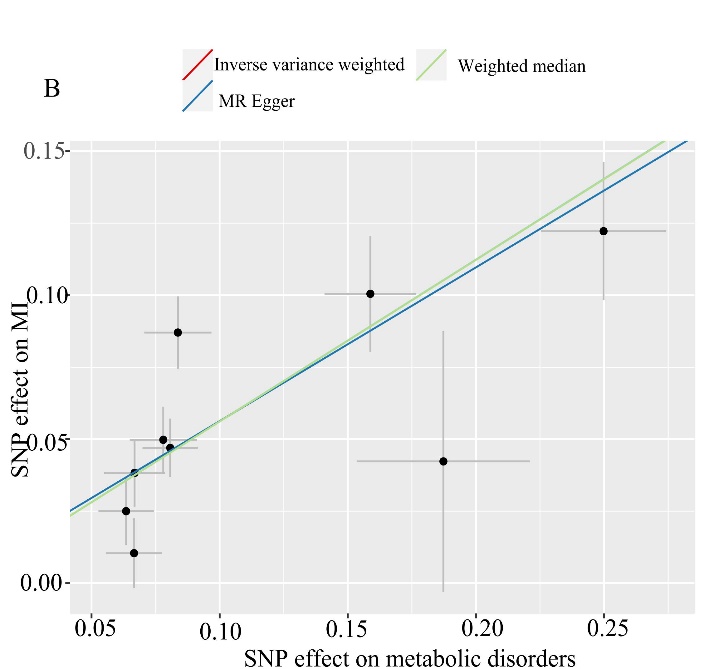

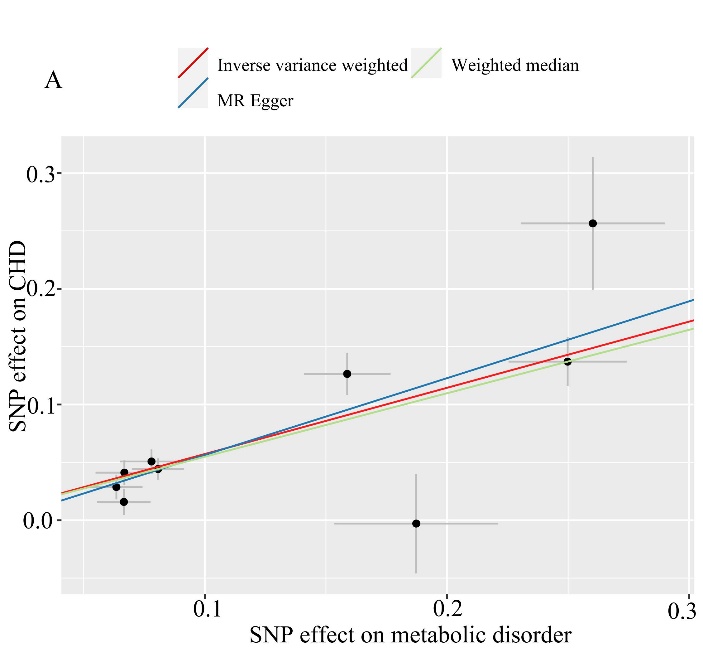


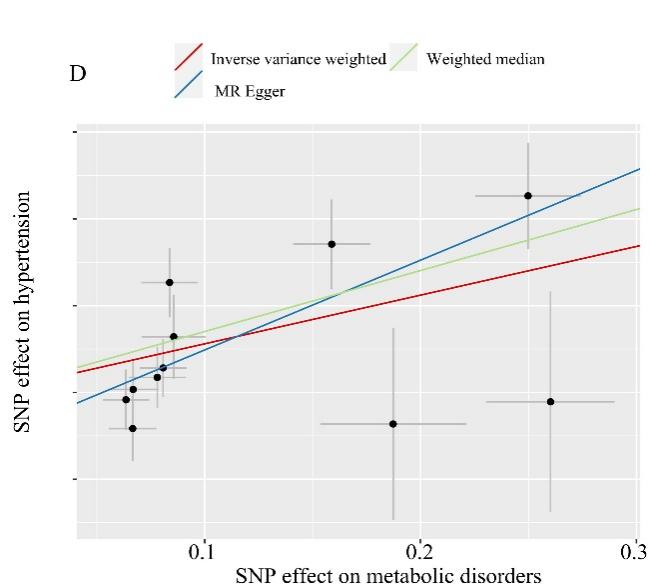

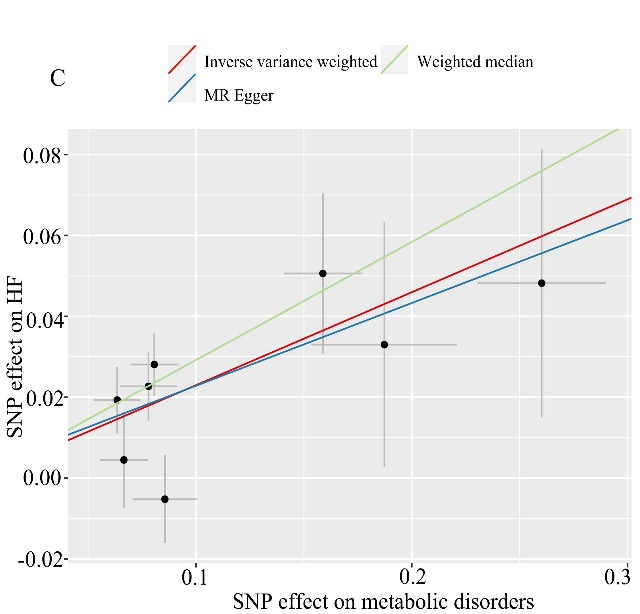


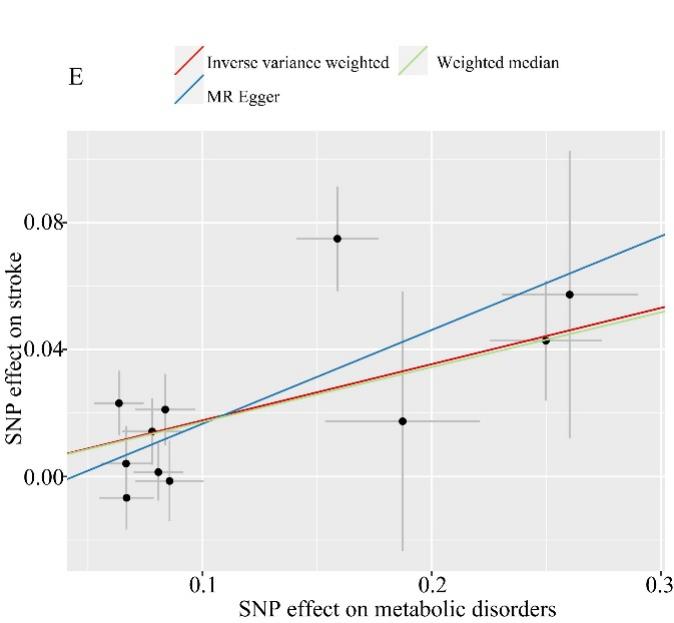


**Supplementary** **Figure 1.** Scatter plot of the association of metabolic disorders with cardiovascular diseases. (A) CHD; (B) MI; (C) HF; (D) hypertension; (E) stroke. CHD, coronary heart disease; MI, myocardial infarction; HF, heart failure; SNP, single-nucleotide polymorphism.


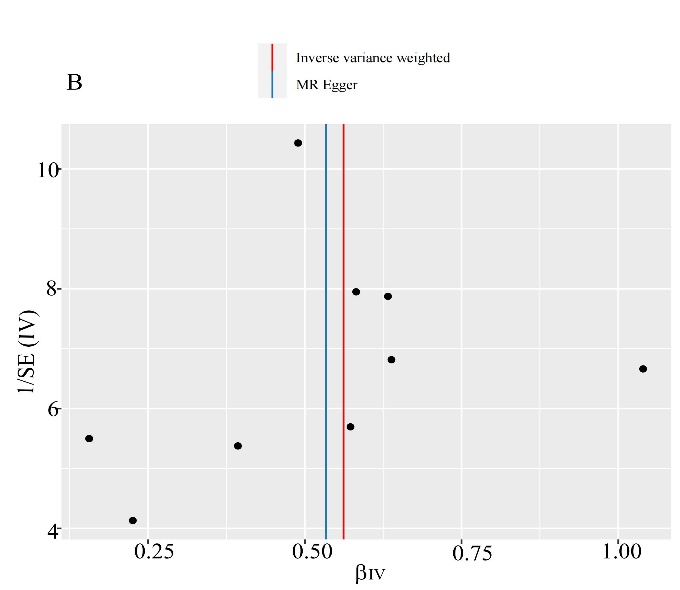

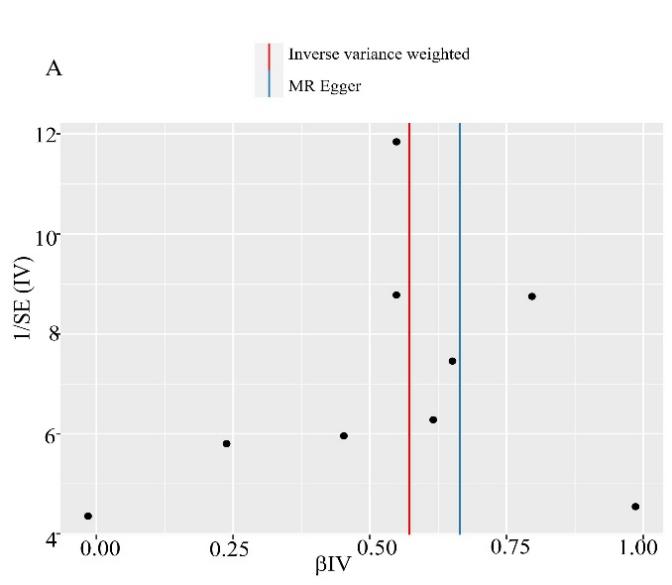


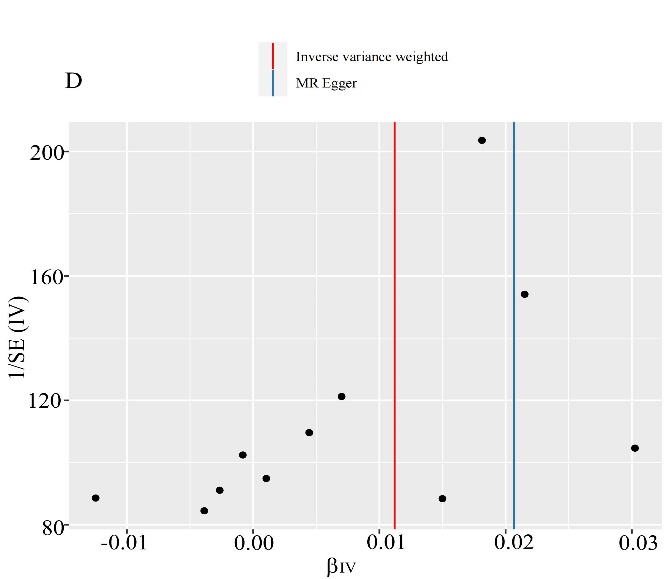

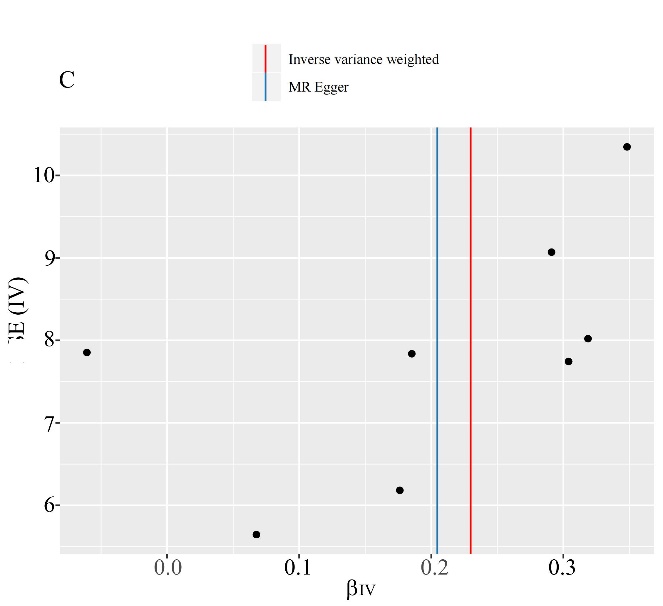


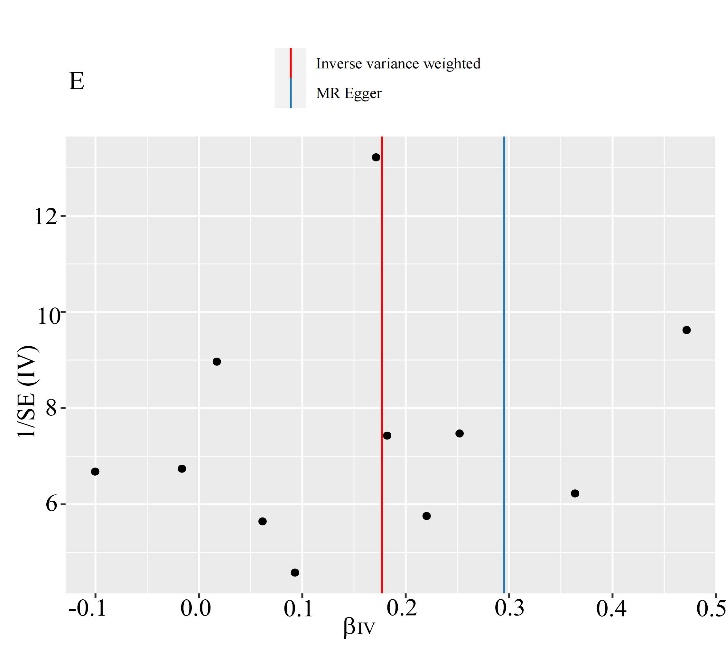


**Supplementary Figure 2.** Funnel plots of the association of metabolic disorders with cardiovascular diseases. (A) CHD; (B) MI (B); (C) HF; (D) hypertension; (E) stroke. CHD, coronary heart disease; MI, myocardial infarction; HF, heart failure; SNP, single-nucleotide polymorphism.


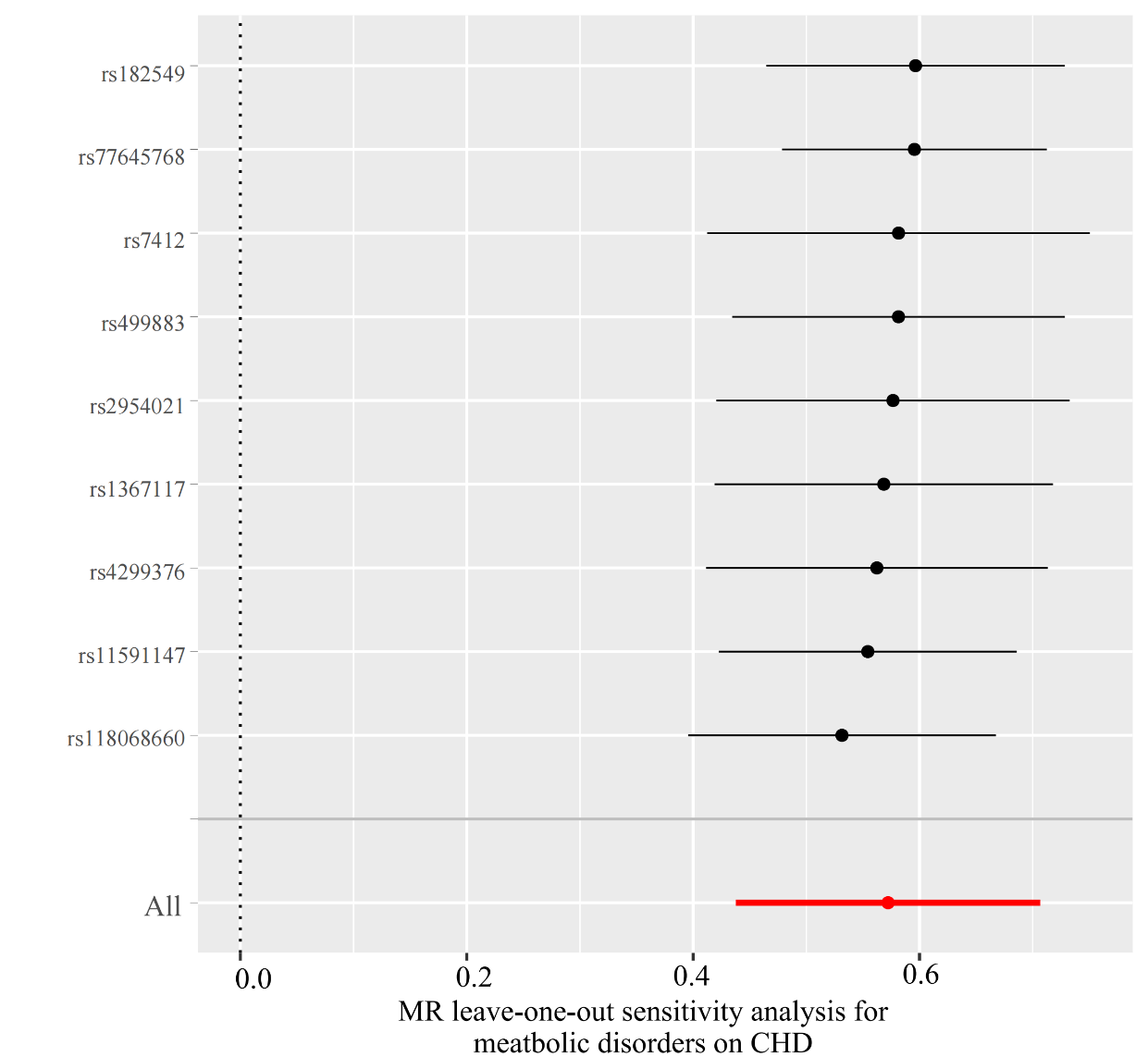


**Supplementary Figure 3.** Leave-one-out sensitivity analysis of the association of

metabolic disorders with CHD. CHD, coronary heart disease.


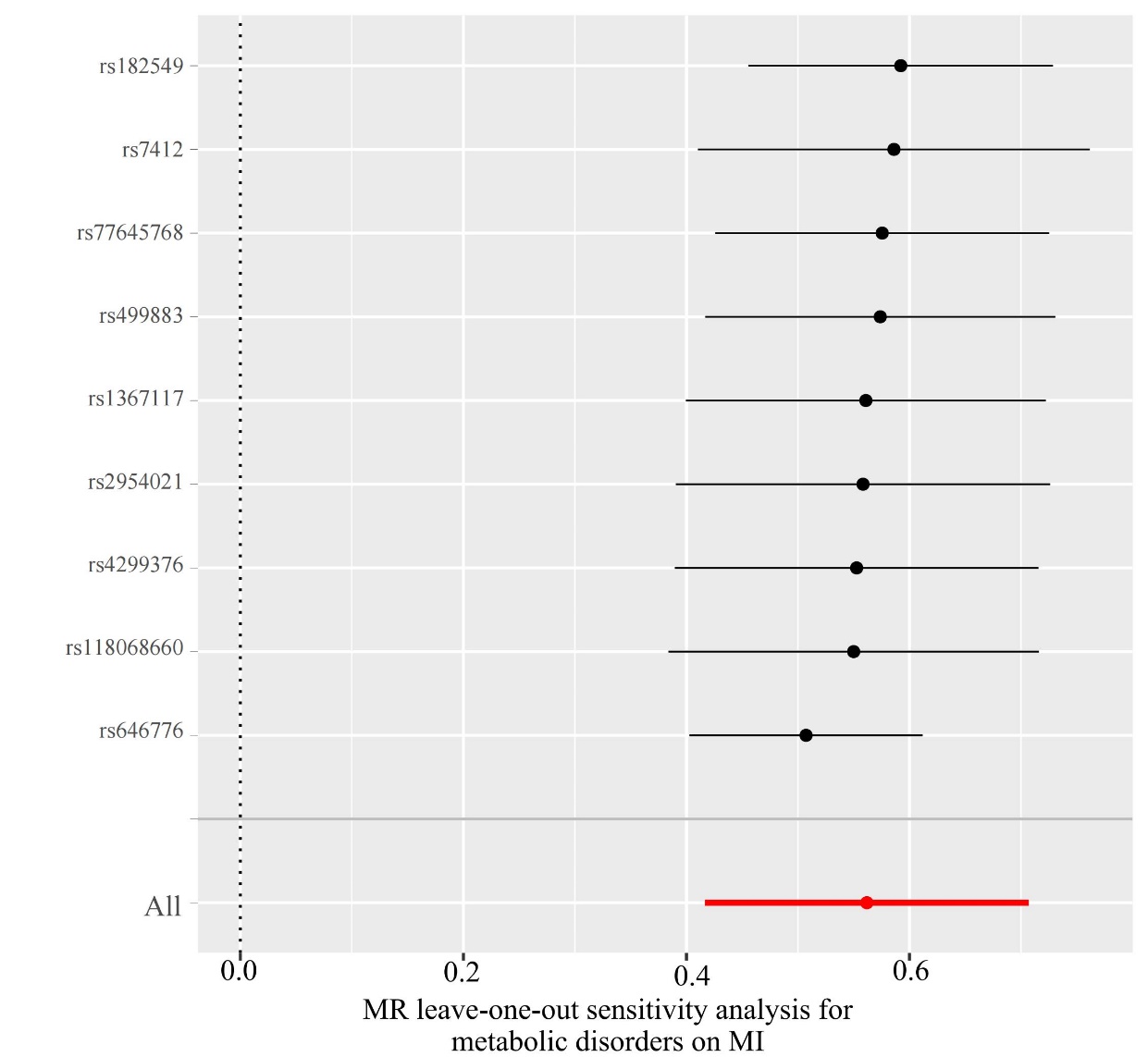


**Supplementary Figure 4.** Leave-one-out sensitivity analysis of the association of

metabolic disorders with MI. MI, myocardial infarction.


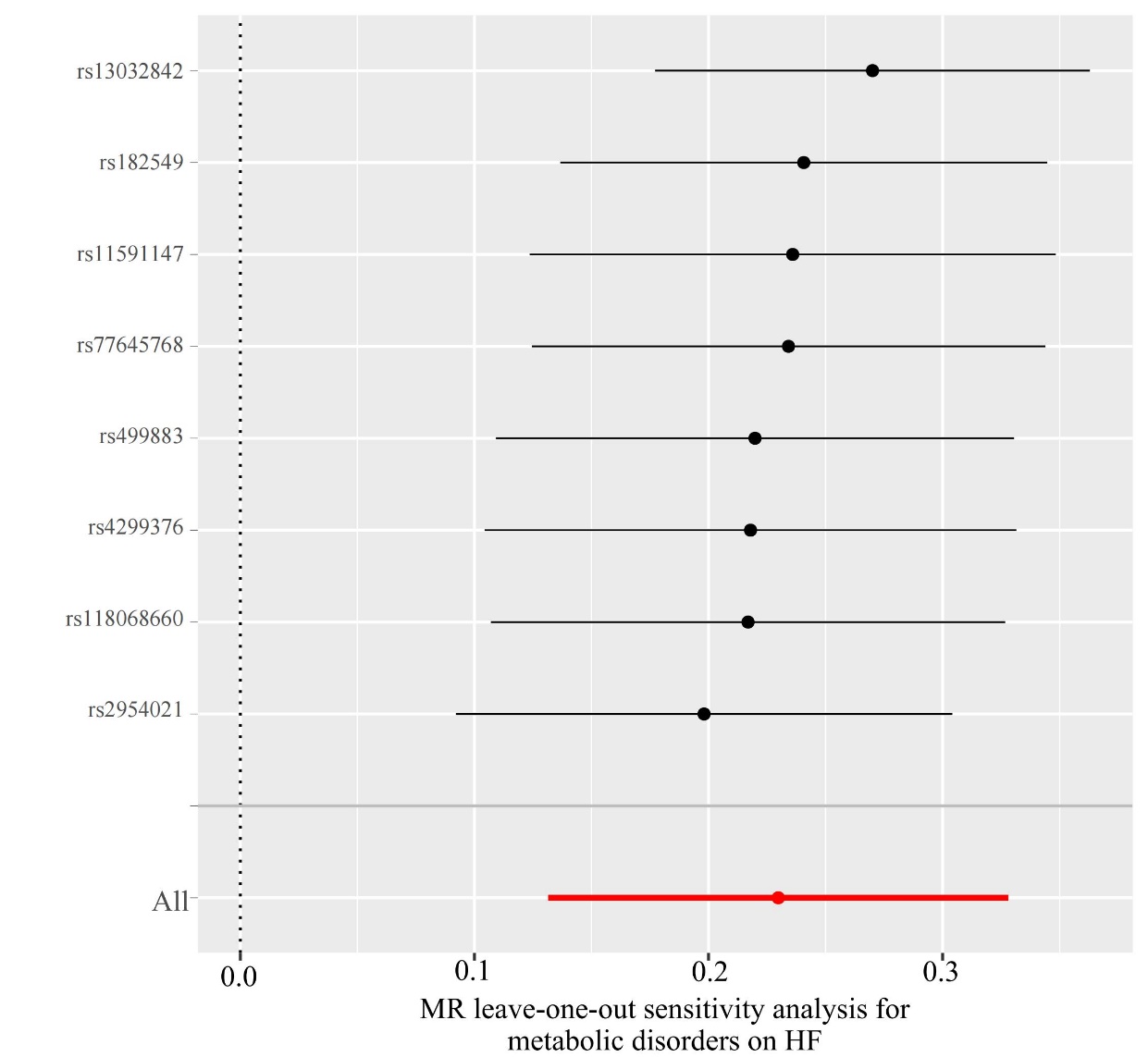


**Supplementary Figure 5.** Leave-one-out sensitivity analysis of the association of

metabolic disorders with HF. HF, heart failure.


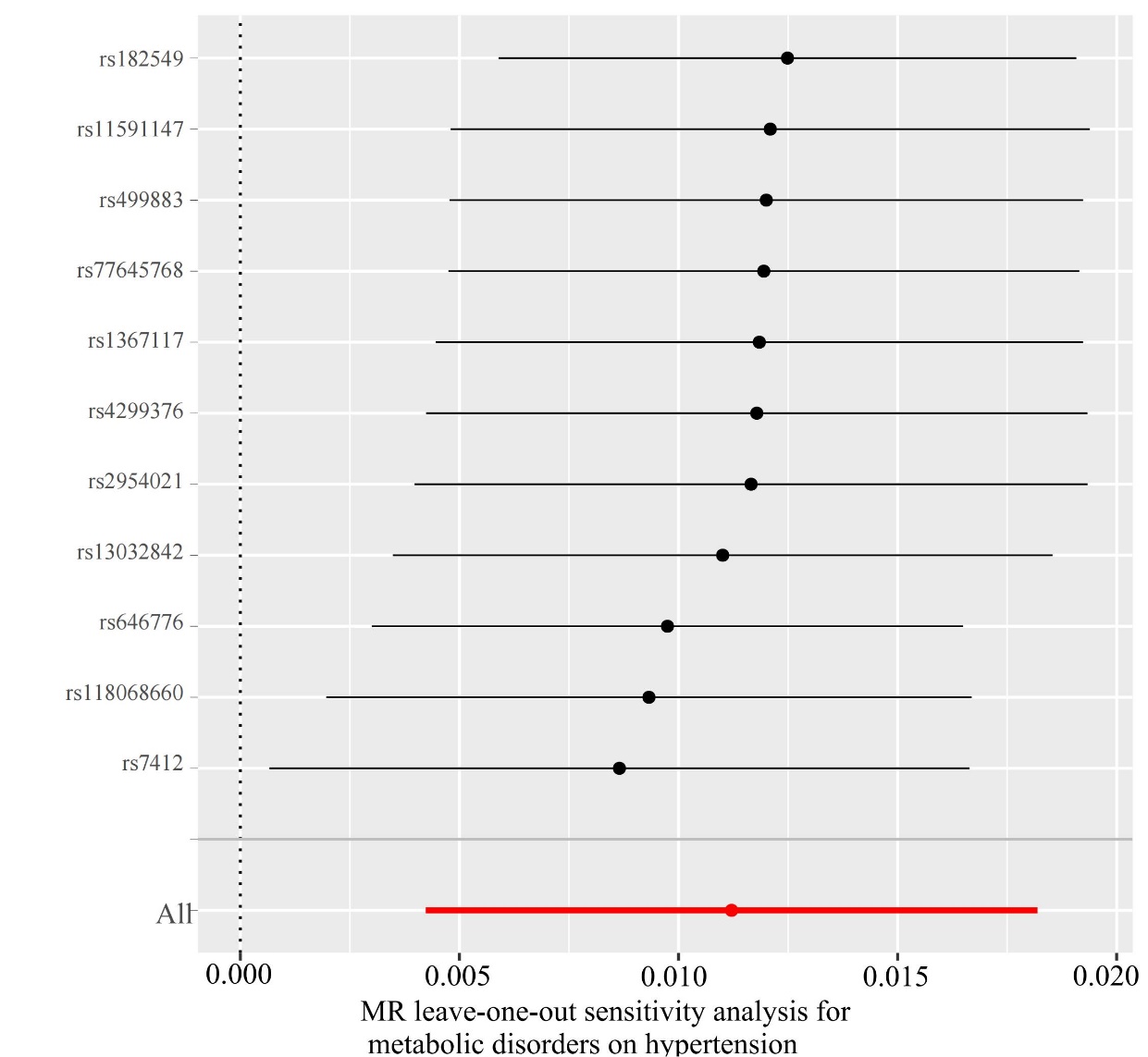


**Supplementary Figure 6.** Leave-one-out sensitivity analysis of the association of

metabolic disorders with hypertension.


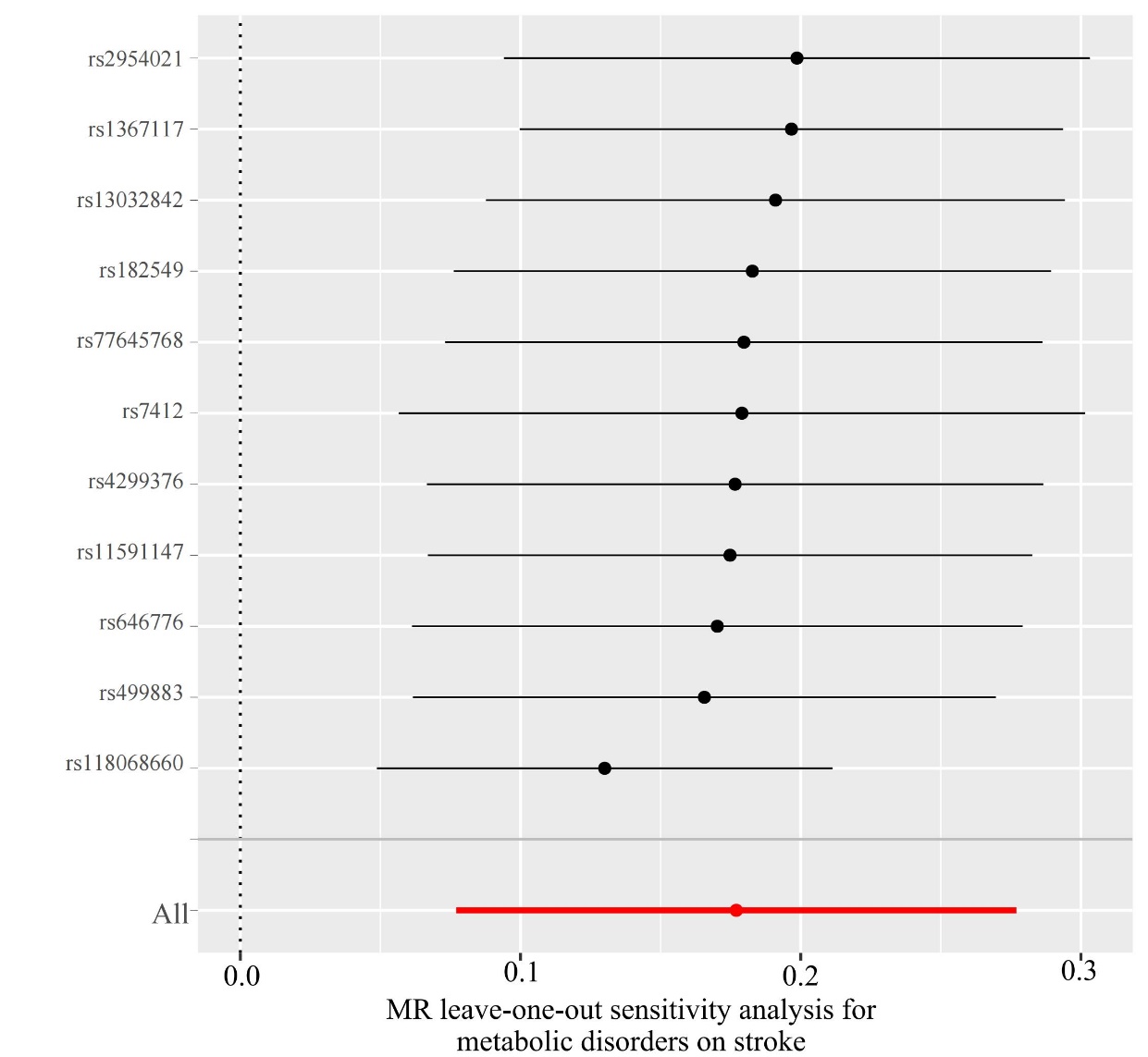


**Supplementary Figure 7.** Leave-one-out sensitivity analysis of the association of

metabolic disorders with stroke.
